# Supplementary material for: Hybrid Models and Biological Model Reduction with PyDSTool
Source: PLoS Comput Biol. 2012 Aug 9;8(8):e1002628. doi: 10.1371/journal.pcbi.1002628 (PMC3415397; doi:10.1371/journal.pcbi.1002628)
Supplement: Text S4 — Complete source code for the PyDSTool package (version 0.88.120504). Includes API documentation and help files linking to web pages. This file is identical to the current public release on Sourceforge.net. (ZIP) [file pcbi.1002628.s004.zip › PyDSTool/html/PyDSTool.PyCont.TestFunc-pysrc.html]

xml version="1.0" encoding="ascii"?


PyDSTool.PyCont.TestFunc


| Home | Trees | Indices | Help | PyDSTool |
| --- | --- | --- | --- | --- |

Package PyDSTool ::
Package PyCont ::
Module TestFunc

[hide private]

[frames] | no frames]

# Source Code for Module PyDSTool.PyCont.TestFunc

```
  1  """ Test functions
 
  2  
 
  3      Drew LaMar, March 2006
 
  4  """ 
  5  
 
  6  # ----------------------------------------------------------------------------
 
  7  # Version History:
 
  8  #   February 2007
 
  9  #       Works with SciPy 0.5.2
 
 10  #
 
 11  #   September 2006
 
 12  #       Added Hopf_Eig test function
 
 13  #       Added UserDefinedTestFunc
 
 14  #
 
 15  #   April 2006
 
 16  #       TestFunc now stores curve class C.
 
 17  #       Added DiscreteMap and FixedPointMap classes.
 
 18  #       Added LPC_Det, NS_Det, PD_Det test functions for FP-C
 
 19  #
 
 20  # ----------------------------------------------------------------------------
 
 21  
 
 22  from misc import * 
 23  from PyDSTool.common import args, copy 
 24  
 
 25  from scipy import optimize, linalg 
 26  from numpy import dot as matrixmultiply 
 27  from numpy import array, float, complex, int, float64, complex64, int32, \
 
 28      zeros, divide, subtract, any, argsort, product, Inf, NaN, isfinite, \
 
 29      r_, c_, sign, mod, mat, subtract, divide, transpose, eye, real, imag, \
 
 30      conjugate, shape, reshape, sqrt, random 
 31  from numpy.random import random 
 32  
 
 33  #####
 
 34  _classes = ['Function', 'TestFunc', 'AddTestFunction', 'DiscreteMap', 'FixedPointMap',
 
 35              'BiAltMethod', 'BorderMethod', 'B_Check', 'Fold_Tan',
 
 36              'Fold_Det', 'Fold_Bor', 'Branch_Det', 'Branch_Bor', 'Hopf_Det', 'Hopf_Bor', 'Hopf_Eig',
 
 37              'Hopf_Double_Bor_One', 'Hopf_Double_Bor_Two', 'BT_Fold', 'BT_Hopf', 'BT_Hopf_One',
 
 38              'CP_Fold', 'DH_Hopf', 'GH_Hopf', 'GH_Hopf_One', 'LPC_Det', 'PD_Det', 'NS_Det',
 
 39              'ParTestFunc', 'UserDefinedTestFunc'] 
 40  
 
 41  __all__ = _classes 
 42  #####
 
 43  
 


44 -class Function(object):


45      """ F: R^n --> R^m
 
 46  
 
 47      Note: The function func needs to act on arrays, not numbers. Thus, in the case where n=1, define
 
 48      the function as acting on a 1 dimensional array, i.e. reference x[0] NOT x.  This is so numjac works
 
 49      without having to test cases (most generality).""" 
 50  
 


51 -    def __init__(self, (n, m), func=None, save=False, numpoints=None):


52          if not hasattr(self, 'data'): 
 53              self.data = None 
 54  
 
 55          if not hasattr(self, 'n'): 
 56              self.n, self.m = n, m 
 57  
 
 58          if save and not hasattr(self, 'vals'): 
 59              self.numpoints = numpoints 
 60  
 
 61              if isinstance(m, int): 
 62                  self.vals = zeros((numpoints, m), float) 
 63              else: 
 64                  self.vals = zeros((numpoints,) + m, float) 
 65  
 
 66          if not hasattr(self, 'func') or self.func is None: 
 67              self.func = func 
 68  
 
 69          self.lastval = None 
 70          self.jac = self.diff 
 71          self.hess = self.numhess

 72  
 


73 -    def __getitem__(self, loc):


74          return self.vals[loc]

 75  
 


76 -    def __setitem__(self, loc, vals):


77          self.vals[loc] = vals

 78  
 


79 -    def __call__(self, *cargs):


80          self.lastval = self.func(*cargs) 
 81          return self.lastval

 82  
 


83 -    def findzero(self, X):


84          return optimize.fsolve(self.func, X)

 85  
 


86 -    def diff(self, x0, ind=None):


87          eps = 1e-6 
 88          try: 
 89              n = len(ind) 
 90          except: 
 91              n = self.n 
 92              ind = range(n) 
 93  
 
 94          J = zeros((self.m, n), float) 
 95          for i in range(n): 
 96              ei = zeros(self.n, float) 
 97              ei[ind[i]] = 1.0 
 98              J[:,i] = (self.func(x0+eps*ei)-self.func(x0-eps*ei))/(2*eps) 
 99          return J

100  
 
101  
 


102 -    def numhess(self, x0, ind1=None, ind2=None):


103          """Computes second derivative using 2nd order centered finite difference scheme.
 
104          MAKE MORE EFFICIENT IN FUTURE (i.e. when an index is in both ind1 and ind2)
 
105  
 
106          Thus, for F: R^n ---> R^m, H is an (m,n1,n2) matrix, where n1, n2 are subsets of [1,...,n]
 
107          """ 
108          eps = 1e-3 
109          try: 
110              n1 = len(ind1) 
111          except: 
112              n1 = self.n 
113              ind1 = range(n1) 
114  
 
115          try: 
116              n2 = len(ind2) 
117          except: 
118              n2 = self.n 
119              ind2 = range(n2) 
120  
 
121          H = zeros((self.m,n1,n2), float) 
122          for i in range(n1): 
123              ei = zeros(self.n, float) 
124              ei[ind1[i]] = 1.0 
125              for j in range(n2): 
126                  ej = zeros(self.n, float) 
127                  ej[ind2[j]] = 1.0 
128                  if ind1[i] == ind2[j]: 
129                      H[:,i,j] = (-1*self.func(x0+2*eps*ei) + 16*self.func(x0+eps*ei) - 30*self.func(x0) + \
 
130                          16*self.func(x0-eps*ei) - self.func(x0-2*eps*ei))/(12*eps*eps) 
131                  else: 
132                      H[:,i,j] = (self.func(x0+eps*(ei+ej)) - self.func(x0+eps*(ei-ej)) - \
 
133                          self.func(x0+eps*(ej-ei)) + self.func(x0-eps*(ei+ej)))/(4*eps*eps) 
134          return H

135  
 
136  
 


137 -class TestFunc(Function):


138      """You need to define the function yourself within an inherited class.""" 


139 -    def __init__(self, (n, m), F, C, save=False, numpoints=None):


140          Function.__init__(self, (n, m), func=None, save=save, numpoints=numpoints) 
141  
 
142          if not hasattr(self, "F"): 
143              self.F = F 
144  
 
145          if not hasattr(self, "C"): 
146              self.C = C

147  
 


148 -    def findzero(self, P1, P2, ind):


149          """Uses secant method to find zero of test function.""" 
150  
 
151          X1, V1 = P1 
152          X2, V2 = P2 
153  
 
154          Z1 = copy(X1) 
155          Z2 = copy(X2) 
156  
 
157          self.C._preTestFunc(X1, V1) 
158          T1 = self.func(X1, V1)[ind] 
159  
 
160          self.C._preTestFunc(X2, V2) 
161          T2 = self.func(X2, V2)[ind] 
162  
 
163          for i in range(self.C.MaxTestIters): 
164              try: 
165                  r = abs(T1/(T1-T2)) 
166                  if r >= 1: 
167                      r = 0.5 
168              except: 
169                  r = 0.5 
170  
 
171              X = X1 + r*(X2-X1) 
172              V = V1 + r*(V2-V1) 
173  
 
174              self.C.Corrector(X,V) 
175  
 
176              self.C._preTestFunc(X, V) 
177              T = self.func(X, V)[ind] 
178  
 
179              if abs(T) < self.C.TestTol and min(linalg.norm(X-X1),linalg.norm(X-X2)) < self.C.VarTol: 
180                  break 
181              else: 
182                  if sign(T) == sign(T2): 
183                      X2 = X 
184                      V2 = V 
185                      T2 = T 
186                  else: 
187                      X1 = X 
188                      V1 = V 
189                      T1 = T 
190  
 
191          if self.C.verbosity >= 2 and i == self.C.MaxTestIters-1: 
192              print 'Maximum test function iterations reached.\n' 
193  
 
194          return X, V

195  
 


196 -    def diff(self, x0, ind=None):


197          """This is recreated from Function class above for TestFunc class.  Why, you ask?  Because for
 
198          test functions, the jacobian of the parent function F needs to be computed before every test
 
199          function call.  Unforunately, this slows things down, but that is unavoidable.""" 
200          eps = 1e-6 
201          try: 
202              n = len(ind) 
203          except: 
204              n = self.n 
205              ind = range(n) 
206  
 
207          J = zeros((self.m, n), float) 
208          for i in range(n): 
209              ei = zeros(self.n, float) 
210              ei[ind[i]] = 1.0 
211  
 
212              self.C._preTestFunc(x0+eps*ei, None) 
213              F1 = self.func(x0+eps*ei, None) 
214  
 
215              self.C._preTestFunc(x0-eps*ei, None) 
216              F2 = self.func(x0-eps*ei, None) 
217  
 
218              J[:,i] = (F1-F2)/(2*eps) 
219          return J

220  
 
221  
 


222 -class BorderMethod(TestFunc):


223      """Border method:
 
224  
 
225              [A   B][V] = [0]
 
226              [C^T D][G]   [1]
 
227  
 
228          where r = corank and:
 
229              A = (n,m)
 
230              s = max(n,m)
 
231              p = s-m+r, q = s-n+r
 
232              B = (n,p)
 
233              C = (m,q)
 
234              D = 0_(q,p)
 
235              V = (m,q)
 
236              G = (p,q)
 
237              0 = (n,q)
 
238              1 = (q,q)
 
239  
 
240          F:R^nm --> R^pq,  F(A) = G
 
241  
 
242          It can also be written as:
 
243  
 
244              [W^T G][A   B] = [0 1]
 
245                     [C^T D]
 
246  
 
247          where now:
 
248              W = (n,p)
 
249              0 = (p,m)
 
250              1 = (p,p)
 
251  
 
252          This is important for calculating derivatives:
 
253  
 
254              S_{z} = -W^T x A_{z} x V
 
255  
 
256          This is implemented in the diff method.
 
257  
 
258          In order to use the Function class, the matrices are put into vector form.
 
259  
 
260      """ 
261  
 


262 -    def __init__(self, (a,b), (n,m), F, C, r=1, update=False, corr=False, save=False, numpoints=None):


263          """F: R^a --> R^b
 
264  
 
265          Note: I did not assign b automatically (although I could - it would just be b = p*q) since
 
266          you may not want to use all of the entries of S.  Some bordering methods are not
 
267          minimally augmented systems and thus only require certain entries of S.""" 
268  
 
269          TestFunc.__init__(self, (a,b), F, C, save=save, numpoints=numpoints) 
270  
 
271          self.update = update 
272          self.corr = corr 
273  
 
274          s = max(n,m) 
275  
 
276          if self.data is None: 
277              self.data = args() 
278          self.data.n, self.data.m = n, m 
279          self.data.p = s-m+r 
280          self.data.q = s-n+r

281  
 


282 -    def setdata(self, A):


283          """Note: p, q <= min(n,m)""" 
284          self.data.Brand = 2*(random((A.shape[0],self.data.p))-0.5) 
285          self.data.Crand = 2*(random((A.shape[1],self.data.q))-0.5) 
286          self.data.D = zeros((self.data.q,self.data.p), float) 
287  
 
288          if self.update: 
289              U, S, Vh = linalg.svd(A) 
290              self.data.B = U[:,-1*self.data.p:] 
291              self.data.C = transpose(Vh)[:,-1*self.data.q:] 
292          else: 
293              self.data.B = self.data.Brand 
294              self.data.C = self.data.Crand

295              #self.data.B = zeros(self.data.Brand.shape, float)
 
296              #self.data.C = zeros(self.data.Crand.shape, float)
 
297              #self.data.B[0,0] = self.data.C[0,0] = self.data.B[1,1] = self.data.C[1,1] = 1.0
 
298  
 


299 -    def updatedata(self, A):


300          if self.update: 
301              if self.corr: 
302                  self.data.B = self.data.w*(linalg.norm(A,1)/linalg.norm(self.data.w,1)) 
303                  self.data.C = self.data.v*(linalg.norm(A,Inf)/linalg.norm(self.data.v,1)) 
304              else: 
305                  # Note: Problem when singular vectors switch smallest singular value (See NewLorenz).
 
306                  #       To overcome this, I have implemented a 1e-8 random nudge.
 
307                  try: 
308                      ALU = linalg.lu_factor(A) 
309                      BC = linalg.lu_solve(ALU, c_[linalg.lu_solve(ALU, self.data.B + 1e-8*self.data.Brand), \
 
310                                           self.data.C + 1e-8*self.data.Crand], trans=1) 
311                      C = linalg.lu_solve(ALU, BC[:,-1*self.data.q:]) 
312                      B = BC[:,0:self.data.p] 
313                  except: 
314                      if self.C.verbosity >= 1: 
315                          print 'Warning: Problem updating border vectors.  Using svd...' 
316                      U, S, Vh = linalg.svd(A) 
317                      B = U[:,-1*self.data.p:] 
318                      C = transpose(Vh)[:,-1*self.data.q:] 
319  
 
320                  bmult = cmult = 1 
321                  if matrixmultiply(transpose(self.data.B), B) < 0: 
322                      bmult = -1 
323                  if matrixmultiply(transpose(self.data.C), C) < 0: 
324                      cmult = -1 
325                  self.data.B = bmult*B*(linalg.norm(A,1)/linalg.norm(B)) 
326                  self.data.C = cmult*C*(linalg.norm(A,Inf)/linalg.norm(C))

327  
 


328 -    def func(self, A):


329          V, W = self.getVW(A) 
330  
 
331          self.data.v = V[0:self.data.m,:] 
332          self.data.w = W[0:self.data.n,:] 
333          self.data.g = V[-1*self.data.p:,:] 
334  
 
335          self.updatedata(A) 
336  
 
337          return self.data.g

338  
 


339 -    def getVW(self, A):


340          # V --> m, W --> n
 
341          #print self.data
 
342          MLU = linalg.lu_factor(c_[r_[A,transpose(self.data.C)], r_[self.data.B,self.data.D]]) 
343          V = linalg.lu_solve(MLU,r_[zeros((self.data.n,self.data.q), float), eye(self.data.q)]) 
344          W = linalg.lu_solve(MLU,r_[zeros((self.data.m,self.data.p), float), eye(self.data.p)],trans=1) 
345  
 
346          return V, W

347  
 


348 -class BiAltMethod(TestFunc):


349 -    def __init__(self, (n,m), F, C, save=False, numpoints=None):


350          TestFunc.__init__(self, (n,m), F, C, save=save, numpoints=numpoints) 
351  
 
352          if self.data is None: 
353              self.data = args() 
354          n = len(self.F.coords) 
355          self.data.P = zeros((n*(n-1)/2, n*(n-1)/2), float)

356  
 


357 -    def bialtprod(self, A, B):


358          n = A.shape[0] 
359          for p in range(1,n): 
360              for q in range(p): 
361                  for r in range(1,n): 
362                      for s in range(r): 
363                          self.data.P[p*(p-1)/2 + q][r*(r-1)/2 + s] = 0.5*(A[p][r]*B[q][s] - A[p][s]*B[q][r] + \
 
364                                                                           B[p][r]*A[q][s] - B[p][s]*A[q][r]) 
365          return self.data.P

366  
 


367 -    def bialtprodeye(self, A):


368          n = A.shape[0] 
369          for p in range(1,n): 
370              for q in range(p): 
371                  for r in range(1,n): 
372                      for s in range(r): 
373                          if r == q: 
374                              self.data.P[p*(p-1)/2 + q][r*(r-1)/2 + s] = -1*A[p][s] 
375                          elif r != p and s == q: 
376                              self.data.P[p*(p-1)/2 + q][r*(r-1)/2 + s] = A[p][r] 
377                          elif r == p and s == q: 
378                              self.data.P[p*(p-1)/2 + q][r*(r-1)/2 + s] = A[p][p] + A[q][q] 
379                          elif r == p and s != q: 
380                              self.data.P[p*(p-1)/2 + q][r*(r-1)/2 + s] = A[q][s] 
381                          elif s == p: 
382                              self.data.P[p*(p-1)/2 + q][r*(r-1)/2 + s] = -1*A[q][r] 
383                          else: 
384                              self.data.P[p*(p-1)/2 + q][r*(r-1)/2 + s] = 0 
385          return self.data.P

386  
 


387 -class AddTestFunction(Function):


388      """Only works with testfuncs that don't have PreTestFunc and rely only on sysfunc.""" 


389 -    def __init__(self, C, TF_type):


390          self.sysfunc = C.sysfunc 
391          self.testfunc = TF_type(self.sysfunc, C) 
392  
 
393          self.coords = self.sysfunc.coords 
394          self.params = self.sysfunc.params 
395  
 
396          Function.__init__(self, (self.sysfunc.n, self.sysfunc.m + self.testfunc.m))

397  
 


398 -    def setdata(self, X, V):


399          if hasattr(self.testfunc, "setdata"): 
400              self.sysfunc.J_coords = self.sysfunc.jac(X, self.sysfunc.coords) 
401              self.sysfunc.J_params = self.sysfunc.jac(X, self.sysfunc.params) 
402              self.testfunc.setdata(X, V)

403  
 


404 -    def func(self, X):


405          self.sysfunc.J_coords = self.sysfunc.jac(X, self.sysfunc.coords) 
406          self.sysfunc.J_params = self.sysfunc.jac(X, self.sysfunc.params) 
407          tmp1 = self.sysfunc(X) 
408          tmp2 = self.testfunc(X, None) 
409          # Broke with move to SciPy 0.5.2
 
410          return c_[[self.sysfunc(X)], [self.testfunc(X, None)]][0]

411  
 


412 -    def diff(self, X, ind=None):


413          return r_[self.sysfunc.jac(X, ind), self.testfunc.jac(X, ind)]

414  
 


415 -class DiscreteMap(Function):


416      """Turns a function into a map composed with itself period times.  Chain rule gives jacobian.
 
417      Note that F: R^n+m --> R^n, where m is the number of free parameters.  Thus, we transform to
 
418      G: R^n+m --> R^n+m given by G(x,p) = [F(x,p), p], where x is state variable and p parameters.
 
419      This gives
 
420  
 
421          DG = [ DF_x DF_p ]
 
422               [ 0    I    ]
 
423  
 
424      Chain rule on F gives DF^n = DF(F^n-1)*DF(F^n-2)*...*DF(F)*DF(X).  Chain rule on G gives
 
425      the same thing as F, and when you keep track of the upper left and upper right blocks of DG composed
 
426      with itself, you arrive at
 
427  
 
428          DF^n_x = DF_x(F^n-1)*DF_x(F^n-2)*...*DF_x(F)*DF_x(X)
 
429          DF^n_a = DF_x(F^n-1)*DF_a(F^n-2) + DF_a(F^n-1) (defined recursively with F^0 = X, F^-1 = 0)
 
430      """ 


431 -    def __init__(self, F, period=1):


432          self.F = F 
433  
 
434          Function.__init__(self, (self.F.n, self.F.m)) 
435          self.period = period

436  
 


437 -    def func(self, X):


438          FX = self.F(X) 
439          if self.period >= 2: 
440              for k in range(2,self.period+1): 
441                  FX = self.F(c_[[FX], [X[self.F.params]]][0]) 
442          return FX

443  
 


444 -    def diff(self, X, ind=None):


445          try: 
446              n = len(ind) 
447          except: 
448              n = self.n 
449              ind = range(n) 
450  
 
451          F_n = zeros((self.period, self.n), float) 
452          F_n[0] = X 
453          for k in range(1,self.period): 
454              F_n[k] = c_[[self.F(F_n[k-1])], [X[self.F.params]]][0] 
455  
 
456          xslice = slice(self.F.coords[0], self.F.coords[-1]+1, 1) 
457          pslice = slice(self.F.params[0], self.F.params[-1]+1, 1) 
458          J = self.F.jac(F_n[0]) 
459          for k in range(1,self.period): 
460              J2 = self.F.jac(F_n[k]) 
461              J[:,xslice] = matrixmultiply(J2[:,xslice] ,J[:,xslice]) 
462              J[:,pslice] = matrixmultiply(J2[:,xslice], J[:,pslice]) + J2[:,pslice] 
463  
 
464          return J[:, ind[0]:ind[-1]+1]

465  
 


466 -class FixedPointMap(Function):


467      """Turns a discrete map into a fixed point map.""" 


468 -    def __init__(self, F):


469          self.F = F 
470  
 
471          Function.__init__(self, (self.F.n, self.F.m))

472  
 


473 -    def func(self, X):


474          return self.F(X) - X[0:self.F.m]

475  
 


476 -    def diff(self, X, ind=None):


477          try: 
478              n = len(ind) 
479          except: 
480              n = self.n 
481              ind = range(n) 
482  
 
483          return self.F.jac(X, ind) - eye(self.F.m, self.F.n)[:,ind[0]:ind[-1]+1]

484  
 
485  # BOUNDARY POINTS
 
486  
 


487 -class B_Check(TestFunc):


488      """There is an attempt here to be a little efficient.  Instead of having a test
 
489      function for every variable and free parameter, just have one test function that
 
490      monitors when one of the boundaries is crossed (self.all).  Once this is triggered,
 
491      find the var/par that crossed and locate using that specific var/par (self.one).""" 


492 -    def __init__(self, F, C, save=False, numpoints=None):


493          TestFunc.__init__(self, (F.n, 1), F, C, save=save, numpoints=numpoints) 
494          # Get the domain for the parameters and variables
 
495          # We want to make sure we stay in these bounds
 
496          lower = {} 
497          upper = {} 
498          for par in C.freepars: 
499              if C.curvetype == 'UD-C': 
500                  if hasattr(C, '_userdomain') and par in C._userdomain.keys(): 
501                      domain = C._userdomain[par] 
502                  else: 
503                      domain = [-1*Inf, Inf] 
504              else: 
505                  domain = C.gensys.query('pardomains')[par] #pdomain[par] 
506              lower[par] = domain[0] 
507              upper[par] = domain[1] 
508  
 
509          for par in C.varslist: 
510              if C.curvetype == 'UD-C': 
511                  if hasattr(C, '_userdomain') and par in C._userdomain.keys(): 
512                      domain = C._userdomain[par] 
513                  else: 
514                      domain = [-1*Inf, Inf] 
515              else: 
516                  domain = C.gensys.query('vardomains')[par] #xdomain[par] 
517              lower[par] = domain[0] 
518              upper[par] = domain[1] 
519  
 
520          for par in C.auxpars: 
521              lower[par] = -1*Inf 
522              upper[par] = Inf 
523  
 
524          # We can compare a row of values to the two following arrays
 
525          # to determine if we are in bounds.
 
526          self.lower = tocoords(C,lower) 
527          self.upper = tocoords(C,upper) 
528  
 
529          self.func = self.all 
530          self.ind = None

531  
 


532 -    def one(self, X, V):


533          return array((X[self.ind] - self.lower[self.ind])*(self.upper[self.ind] - X[self.ind]))

534  
 


535 -    def all(self, X, V):


536          return array((any(X < self.lower) or any(X > self.upper)) and -1 or 1)

537  
 
538  # BRANCH POINTS
 
539  
 


540 -class Branch_Det(TestFunc):


541 -    def __init__(self, F, C, save=False, numpoints=None):


542          TestFunc.__init__(self, (F.n, 1), F, C, save=save, numpoints=numpoints)

543  
 


544 -    def func(self, X, V):


545          return array([linalg.det(r_[c_[self.F.J_coords,self.F.J_params],[V]])])

546  
 


547 -class Branch_Bor(BorderMethod):


548 -    def __init__(self, F, C, update=True, save=False, numpoints=None):


549          BorderMethod.__init__(self, (F.n, 2), (F.n-1,F.n), F, C, update=update, save=save, numpoints=numpoints)

550  
 


551 -    def setdata(self, X, V):


552          BorderMethod.setdata(self, c_[self.F.J_coords,self.F.J_params])

553  
 


554 -    def func(self, X, V):


555          return array(BorderMethod.func(self, c_[self.F.J_coords,self.F.J_params])[0])

556  
 
557  # FOLD POINTS
 
558  
 


559 -class Fold_Det(TestFunc):


560 -    def __init__(self, F, C, save=False, numpoints=None):


561          TestFunc.__init__(self, (F.n, 1), F, C, save=save, numpoints=numpoints)

562  
 


563 -    def func(self, X, V):


564          return array([linalg.det(self.F.J_coords)])

565  
 


566 -class Fold_Tan(TestFunc):


567 -    def __init__(self, F, C, save=False, numpoints=None):


568          TestFunc.__init__(self, (F.n, 1), F, C, save=save, numpoints=numpoints)

569  
 


570 -    def func(self, X, V):


571          return array([V[-1]])

572  
 


573 -class Fold_Bor(BorderMethod):


574 -    def __init__(self, F, C, update=True, corr=True, save=False, numpoints=None):


575          BorderMethod.__init__(self, (F.n, 1), (F.m,F.m), F, C, update=update, corr=corr, save=save, numpoints=numpoints)

576  
 


577 -    def setdata(self, X, V):


578          BorderMethod.setdata(self, self.F.J_coords)

579  
 


580 -    def func(self, X, V):


581          return array(BorderMethod.func(self, self.F.J_coords)[0])

582  
 


583 -    def diff(self, X, ind=None):


584          try: 
585              n = len(ind) 
586          except: 
587              n = self.n 
588              ind = range(n) 
589  
 
590          V, W = self.getVW(self.F.jac(X, self.F.coords)) 
591          H = self.F.hess(X, self.F.coords, ind) 
592          return -1*reshape([bilinearform(H[:,:,i], V[0:self.data.m,:], W[0:self.data.n,:]) \
 
593              for i in range(n)],(1,len(ind)))

594  
 
595  # HOPF POINTS
 
596  
 


597 -class Hopf_Det(BiAltMethod):


598 -    def __init__(self, F, C, save=False, numpoints=None):


599          BiAltMethod.__init__(self, (F.n,1), F, C, save=save, numpoints=numpoints)

600  
 


601 -    def func(self, X, V):


602          self.bialtprodeye(2*self.F.J_coords) 
603          return array([linalg.det(self.data.P)])

604  
 


605 -class Hopf_Bor(BorderMethod, BiAltMethod):


606 -    def __init__(self, F, C, update=True, corr=True, save=False, numpoints=None):


607          n = F.m 
608          BiAltMethod.__init__(self, (F.n, 1), F, C, save=save, numpoints=numpoints) 
609          BorderMethod.__init__(self, (F.n, 1), (n*(n-1)/2, n*(n-1)/2), F, C, update=update, corr=corr, save=save, numpoints=numpoints)

610  
 


611 -    def setdata(self, X, V):


612          self.bialtprodeye(2*self.F.J_coords) 
613          BorderMethod.setdata(self, self.data.P)

614  
 


615 -    def func(self, X ,V):


616          self.bialtprodeye(2*self.F.J_coords) 
617          return array(BorderMethod.func(self, self.data.P)[0])

618  
 


619 -    def diff(self, X, ind=None):


620          try: 
621              n = len(ind) 
622          except: 
623              n = self.n 
624              ind = range(n) 
625  
 
626          self.bialtprodeye(2*self.F.jac(X, self.F.coords)) 
627          V, W = self.getVW(self.data.P) 
628          H = self.F.hess(X, self.F.coords, ind) 
629          return -1*reshape([bilinearform(self.bialtprodeye(2*H[:,:,i]), V[0:self.data.m,:], W[0:self.data.n,:]) \
 
630              for i in range(n)],(1,len(ind)))

631  
 


632 -class Hopf_Eig(TestFunc):


633 -    def __init__(self, F, C, save=False, numpoints=None):


634          TestFunc.__init__(self, (F.n, 1), F, C, save=save, numpoints=numpoints) 
635          self.sgnnum = 0 
636          self.sgn = 1

637  
 


638 -    def func(self, X, V):


639          eigs, LV = linalg.eig(self.F.J_coords) 
640          reigs = [real(eig) for eig in eigs if abs(imag(eig)) >= 0.0] 
641          sgnnum = len([z for z in reigs if z < 0.0]) 
642          if self.sgnnum != sgnnum: 
643              self.sgnnum = sgnnum 
644              self.sgn = -1*self.sgn 
645          return array([self.sgn*min([abs(r) for r in reigs])])

646  
 
647  # Codimension-1 continuation
 
648  
 


649 -class Hopf_Double_Bor_One(BorderMethod, BiAltMethod):


650 -    def __init__(self, F, C, update=False, save=False, numpoints=None):


651          n = F.m 
652          BiAltMethod.__init__(self, (F.n,1), F, C, save=save, numpoints=numpoints) 
653          BorderMethod.__init__(self, (F.n,1), (n*(n-1)/2, n*(n-1)/2), F, C, r=2, corr=True, update=update, save=save, numpoints=numpoints)

654  
 


655 -    def setdata(self, X, V):


656          A = self.bialtprodeye(2*self.F.J_coords) 
657          """Note: p, q <= min(n,m)""" 
658  
 
659          self.data.Brand = 2*(random((A.shape[0],self.data.p))-0.5) 
660          self.data.Crand = 2*(random((A.shape[1],self.data.q))-0.5) 
661          self.data.B = zeros((A.shape[0],self.data.p), float) 
662          self.data.C = zeros((A.shape[1],self.data.q), float) 
663          self.data.D = zeros((self.data.q,self.data.p), float) 
664  
 
665          U, S, Vh = linalg.svd(A) 
666          self.data.b = U[:,-1:] 
667          self.data.c = transpose(Vh)[:,-1:] 
668  
 
669          if self.update: 
670              self.data.B[:,1] = self.data.b 
671              self.data.C[:,1] = self.data.c 
672  
 
673              U2, S2, Vh2 = linalg.svd(c_[r_[A, transpose(self.data.C[:,1])], r_[self.data.B[:,1], [[0]]]]) 
674              self.data.B[:,2] = U2[0:A.shape[0],-1:] 
675              self.data.C[:,2] = transpose(Vh2)[0:A.shape[1],-1:] 
676              self.data.D[0,1] = U2[A.shape[0],-1] 
677              self.data.D[1,0] = transpose(Vh2)[A.shape[1],-1] 
678          else: 
679              self.data.B = self.data.Brand 
680              self.data.C = self.data.Crand

681              #self.data.B[0,0] = self.data.C[0,0] = self.data.B[1,1] = self.data.C[1,1] = 1.0
 
682  
 


683 -    def updatedata(self, A):


684          # Update b, c
 
685          try: 
686              ALU = linalg.lu_factor(A) 
687              BC = linalg.lu_solve(ALU, c_[linalg.lu_solve(ALU, self.data.b + 1e-8*self.data.Brand[:,:1]), \
 
688                                   self.data.c + 1e-8*self.data.Crand[:,:1]], trans=1) 
689              C = linalg.lu_solve(ALU, BC[:,-1:]) 
690              B = BC[:,:1] 
691          except: 
692              if self.C.verbosity >= 1: 
693                  print 'Warning: Problem updating border vectors.  Using svd...' 
694              U, S, Vh = linalg.svd(A) 
695              B = U[:,-1:] 
696              C = transpose(Vh)[:,-1:] 
697  
 
698          bmult = cmult = 1 
699          if matrixmultiply(transpose(self.data.b), B) < 0: 
700              bmult = -1 
701          if matrixmultiply(transpose(self.data.c), C) < 0: 
702              cmult = -1 
703          self.data.b = bmult*B*(linalg.norm(A,1)/linalg.norm(B)) 
704          self.data.c = cmult*C*(linalg.norm(A,Inf)/linalg.norm(C)) 
705  
 
706          # Update
 
707          if self.update: 
708              self.data.B[:,0] = self.data.b*(linalg.norm(A,1)/linalg.norm(self.data.b)) 
709              self.data.C[:,0] = self.data.c*(linalg.norm(A,Inf)/linalg.norm(self.data.c)) 
710  
 
711              self.data.B[:,1] = self.data.w[:,2]*(linalg.norm(A,1)/linalg.norm(self.data.w,1)) 
712              self.data.C[:,1] = self.data.v[:,2]*(linalg.norm(A,Inf)/linalg.norm(self.data.v,1)) 
713  
 
714              self.data.D[0,1] = self.data.g[0,1] 
715              self.data.D[1,0] = self.data.g[1,0]

716  
 


717 -    def func(self, X, V):


718          self.bialtprodeye(2*self.F.J_coords) 
719          return array([linalg.det(BorderMethod.func(self, self.data.P))])

720  
 


721 -class Hopf_Double_Bor_Two(BorderMethod):


722      # Define diff at some point
 


723 -    def __init__(self, F, C, update=False, save=False, numpoints=None):


724          BorderMethod.__init__(self, (F.n, 2), (F.m,F.m), F, C, r=2, corr=True, update=update, save=save, numpoints=numpoints)

725  
 


726 -    def setdata(self, X, V):


727          BorderMethod.setdata(self, matrixmultiply(self.F.J_coords,self.F.J_coords) + X[-1]*eye(self.F.m))

728  
 


729 -    def func(self, X, V):


730          BorderMethod.func(self, matrixmultiply(self.F.J_coords,self.F.J_coords) + X[-1]*eye(self.F.m)) 
731          return array([self.data.g[0,0], self.data.g[1,1]])

732  
 
733      # def diff(self, X, ind=None):
 
734          # try:
 
735              # n = len(ind)
 
736          # except:
 
737              # n = self.n
 
738              # ind = range(n)
 
739  #
 
740          # J_coords = self.F.jac(X, self.F.coords)
 
741          # V, W = self.getVW(matrixmultiply(J_coords,J_coords) + X[-1]*eye(self.F.m))
 
742          # H = self.F.hess(X, self.F.coords, ind)
 
743          # dG = -1*array([bilinearform(H[:,:,i], V[0:self.data.m,:], W[0:self.data.n,:]) \
 
744              # for i in range(n)])
 
745          # #return r_[reshape(dG[:,0,0], (1,len(ind))), reshape(dG[:,1,1], (1,len(ind)))]
 
746          # return reshape(r_[dG[:,0,0], dG[:,1,1]], (2, len(ind)))
 
747  
 
748  # Codimension-2 bifurcations
 
749  
 


750 -class BT_Fold(TestFunc):


751 -    def __init__(self, F, C, save=False, numpoints=None):


752          TestFunc.__init__(self, (F.n, 1), F, C, save=save, numpoints=numpoints)

753  
 


754 -    def func(self, X, V):


755          return array(matrixmultiply(transpose(self.F.testfunc.data.w), self.F.testfunc.data.v)[0])

756  
 


757 -class CP_Fold(TestFunc):


758 -    def __init__(self, F, C, save=False, numpoints=None):


759          TestFunc.__init__(self, (F.n, 1), F, C, save=save, numpoints=numpoints)

760  
 


761 -    def func(self, X, V):


762          H = self.F.sysfunc.hess(X, self.F.coords, self.F.coords) 
763          q = self.F.testfunc.data.v/linalg.norm(self.F.testfunc.data.v) 
764          p = self.F.testfunc.data.w/matrixmultiply(transpose(self.F.testfunc.data.w),q) 
765  
 
766          return array(0.5*matrixmultiply(transpose(p), reshape([bilinearform(H[i,:,:], q, q) \
 
767               for i in range(H.shape[0])],(H.shape[0],1)))[0])

768  
 
769  # Test functions for HopfCurveOne
 
770  
 


771 -class DH_Hopf(TestFunc):


772 -    def __init__(self, F, C, save=False, numpoints=None):


773          TestFunc.__init__(self, (F.n, 1), F, C, save=save, numpoints=numpoints)

774  
 


775 -    def func(self, X, V):


776          return array([self.F.testfunc.data.g[1,1]])

777  
 


778 -class BT_Hopf_One(TestFunc):


779 -    def __init__(self, F, C, save=False, numpoints=None):


780          TestFunc.__init__(self, (F.n, 1), F, C, save=save, numpoints=numpoints)

781  
 


782 -    def func(self, X, V):


783          k = self.C.TFdata.k 
784  
 
785          return array([k])

786  
 


787 -class GH_Hopf_One(TestFunc):


788 -    def __init__(self, F, C, save=False, numpoints=None):


789          TestFunc.__init__(self, (F.n, 1), F, C, save=save, numpoints=numpoints)

790  
 


791 -    def func(self, X, V):


792          k = self.C.TFdata.k 
793          v1 = self.C.TFdata.v1 
794          w1 = self.C.TFdata.w1 
795  
 
796          if k >=0: 
797              J_coords = self.F.sysfunc.J_coords 
798              w = sqrt(k) 
799  
 
800              q = v1 - (1j/w)*matrixmultiply(self.F.sysfunc.J_coords,v1) 
801              p = w1 + (1j/w)*matrixmultiply(transpose(self.F.sysfunc.J_coords),w1) 
802  
 
803              p /= linalg.norm(p) 
804              q /= linalg.norm(q) 
805  
 
806              p = reshape(p,(p.shape[0],)) 
807              q = reshape(q,(q.shape[0],)) 
808  
 
809              direc = conjugate(1/matrixmultiply(transpose(conjugate(p)),q)) 
810              p = direc*p 
811  
 
812              l1 = firstlyapunov(X, self.F.sysfunc, w, J_coords=J_coords, p=p, q=q) 
813  
 
814              return array([l1]) 
815          else: 
816              return array([1])

817  
 
818  # Test functions for HopfCurveTwo
 
819  
 


820 -class BT_Hopf(TestFunc):


821 -    def __init__(self, F, C, save=False, numpoints=None):


822          TestFunc.__init__(self, (F.n, 1), F, C, save=save, numpoints=numpoints)

823  
 


824 -    def func(self, X, V):


825          return array([X[-1]])

826  
 


827 -class GH_Hopf(TestFunc):


828 -    def __init__(self, F, C, save=False, numpoints=None):


829          TestFunc.__init__(self, (F.n, 1), F, C, save=save, numpoints=numpoints)

830  
 


831 -    def func(self, X, V):


832          if X[-1] >=0: 
833              J_coords = self.F.sysfunc.J_coords 
834              w = sqrt(X[-1]) 
835  
 
836              l1 = firstlyapunov(X, self.F.sysfunc, w, J_coords=J_coords, V=self.F.testfunc.data.v, W=self.F.testfunc.data.w) 
837  
 
838              return array([l1]) 
839          else: 
840              return array([1])

841  
 
842  # Test functions for FixedPointCurve
 
843  
 


844 -class LPC_Det(TestFunc):


845 -    def __init__(self, F, C, save=False, numpoints=None):


846          TestFunc.__init__(self, (F.n, 1), F, C, save=save, numpoints=numpoints)

847  
 


848 -    def func(self, X, V):


849          return array([linalg.det(self.F.J_coords - eye(self.F.m, self.F.m))])

850  
 


851 -class PD_Det(TestFunc):


852 -    def __init__(self, F, C, save=False, numpoints=None):


853          TestFunc.__init__(self, (F.n, 1), F, C, save=save, numpoints=numpoints)

854  
 


855 -    def func(self, X, V):


856          return array([linalg.det(self.F.J_coords + eye(self.F.m, self.F.m))])

857  
 


858 -class NS_Det(BiAltMethod):


859 -    def __init__(self, F, C, save=False, numpoints=None):


860          BiAltMethod.__init__(self, (F.n,1), F, C, save=save, numpoints=numpoints)

861  
 


862 -    def func(self, X, V):


863          n = self.F.m*(self.F.m-1)/2 
864          self.bialtprod(self.F.J_coords,self.F.J_coords) 
865          return array([linalg.det(self.data.P - eye(n,n))])

866  
 
867  
 
868  # Test function for stopping at parameter values (SPOut for PyCont)
 
869  
 


870 -class ParTestFunc(TestFunc):


871 -    def __init__(self, n, C, pix, pval, save=False, numpoints=None):


872          TestFunc.__init__(self, (n, 1), C.sysfunc, C, save=save, numpoints=numpoints) 
873          self._pval = pval 
874          self._pix = pix

875  
 


876 -    def func(self, X, V):


877          return array([X[self._pix] - self._pval])

878  
 
879  
 
880  # Test function for UserDefinedCurve
 
881  
 


882 -class UserDefinedTestFunc(TestFunc):


883 -    def __init__(self, (n, m), C, tfunc, save=False, numpoints=None):


884          TestFunc.__init__(self, (n, m), C.sysfunc, C, save=save, numpoints=numpoints) 
885  
 
886          self.tfunc = tfunc

887  
 


888 -    def setdata(self, X, V):


889          self.C._userdata.sgn = -1 
890          self.C._userdata.val = 1

891  
 


892 -    def func(self, X, V):


893          return self.tfunc(self.C._userdata, X, V)

894
```

  


| Home | Trees | Indices | Help | PyDSTool |
| --- | --- | --- | --- | --- |

|  |  |
| --- | --- |
| Generated by Epydoc 3.0.1 on Fri May 4 15:24:11 2012 | http://epydoc.sourceforge.net |
